# LSRR-LA: An Anisotropy-Tolerant Localization Algorithm Based on Least Square Regularized Regression for Multi-Hop Wireless Sensor Networks

**DOI:** 10.3390/s18113974

**Published:** 2018-11-15

**Authors:** Wei Zhao, Fei Shao, Song Ye, Wei Zheng

**Affiliations:** 1 Jiangsu Key Laboratory of Data Science & Smart Software, Jinling Institute of Technology, Nanjing 211169, China; shaofei@jit.edu.cn; 2 School of Computer Engineering, Jinling Institute of Technology, Nanjing 211169, China; ye_song@jit.edu.cn (S.Y.); zhengwei@jit.edu.cn (W.Z.)

**Keywords:** wireless sensor network, multi-hop localization, regression

## Abstract

As is well known, multi-hop range-free localization algorithms demonstrate pretty good performance in isotropic networks in which sensor nodes distribute evenly and densely. However, these algorithms are easily affected by network topology, causing a significant decrease in positioning accuracy. To improve the localization performance in anisotropic networks, this paper presents a multi-hop range-free localization algorithm based on Least Square Regularized Regression (LSRR). By building a mapping relationship between hop counts and real distances, we can regard the process of localization as a regularized regression. Firstly, the proximity information of the given network is measured. Then, a mapping model between the geographical distances and the hop distances is constructed by LSRR. Finally, each sensor node finds its own position via this mapping. The Average Localization Error (ALE) metric is used to evaluate the proposed method in our experiments, and results show that, compared with similar methods, our approach can effectively decrease the effect of anisotropy, thus considerably improving the positioning accuracy.

## 1. Introduction

Obtaining accurate location information is a precondition for many applications that are based on localization technology. Among the existing positioning technologies, GPS satellite positioning systems rely on their global coverage and high accuracy, and have been widely used [1,2] all over the world. However, these satellite positioning systems also have some significant shortcomings, including high energy consumption, a high cost, fixed auxiliary facilities, and the only use of open space. Since the 1990s, Wireless Sensor Networks (WSNs) have attracted the attention of many relevant researchers. As an extension of a satellite positioning system, WSNs bring localization methods a low cost, low power consumption, and no additional auxiliary facilities. In some applications, WSN-based localization systems still work well while satellite positioning systems may be out of service [3,4,5]. For example, WSN localization approaches can provide us with a relatively accurate location in areas where satellite signals are blocked, such as primitive forests and buildings. Some localization methods of WSNs are based on the characteristics of network connectivity and multi-hop information. With the help of known nodes, which are called anchors, the unknown nodes can estimate their locations. Such localization technologies are also called multi-hop range-free localization methods [6,7]. Localization approaches in WSNs can be divided into two types: range-based algorithms [8,9] and range-free algorithms [10,11,12]. The range-based (e.g., RSSI-based, Received Signal Strength Indication) localization algorithms estimate the locations of sensor nodes by measuring their distances or angles from the anchor nodes. The range-based localization algorithms can deal with anisotropic networks and ensure a relatively accurate estimation of sensor locations; however, these algorithms are easily affected by environmental factors, such as noise and obstacles. In addition, their complexity and cost also increase with the accuracy requirement. For a large-scale sensor network with many sensor nodes, it is not reasonable to equip them all with ranging devices. In contrast, range-free localization algorithms use only the connectivity information among nodes, i.e., they do not require expensive ranging devices. In the case of large-scale WSN localization applications, the range-free algorithms would be preferred where sensor nodes are provided with a small amount of resources. Since the multi-hop range-free positioning technique only needs the hop information between nodes, it can position unknown nodes without additional devices and thus has been widely used.

**Figure 1:**
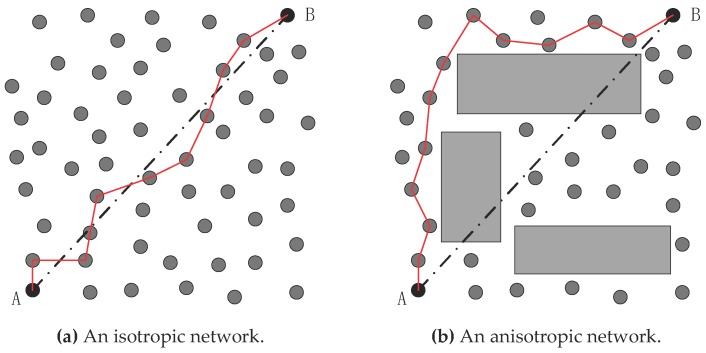
An example of network anisotropy’s impact on the hop distance.

The multi-hop range-free localization methods perform rather well in an even and dense network, which frequently means that a large number of nodes are deployed in a limited area. They seriously depend on the network topology. Taking Figure 1 as an example, the dotted lines indicate the geometric distance between nodes A and B, while the red solid lines denote the corresponding hop distances between them. If the sensor nodes are distributed evenly, the geometric distances will roughly match the hop distances (refer to Figure 1a). However, this matching relationship will be totally undermined if there are some obstacles that bend the paths between nodes A and B, leading to a mismatch between the geometric distances and the hop distances (refer to Figure 1b). We usually call a network like that shown in Figure 1b an anisotropic network, which has different propagation models and irregular localization areas. A large deviation between the geometric distances and hop distances may probably cause a sharp drop in positioning accuracy, which is exactly the reason that the multi-hop range-free localization methods perform so poorly in anisotropic networks.

To improve the positioning accuracy in such anisotropic networks, we propose a multi-hop range-free localization method based on Least Square Regularized Regression, i.e., LSRR-LA (Least Square Regularized Regression based multi-hop range-free Localization Algorithm). In the case of LSRR-LA, we build a mapping model between geometric distances and hop counts by using Least Square Regularized Regression, and then we estimate the locations of unknown nodes in terms of this model. During the establishment step of this mapping model, structural risk minimization is obtained by tuning the weights between the empirical risk and a penalty item, thus ensuring enough stability in, and accuracy of, this mapping model.

The rest of this paper is organized as follows. Related works are briefly introduced in Section 2. Section 3 describes the proposed LSRR-LA method in detail. Extensive simulation experiments are conducted to evaluate the performance of LSRR-LA and make comparisons with typical localization solutions, and the results are reported in Section 4. Finally, Section 5 gives some conclusions.

## 2. Related Works

As far as multi-hop range-free localization algorithms are concerned, some popular ones include DV-hop (Distance Vector hop) [13,14], Amorphous [15], and MDS-MAP (Multidimensional scaling MDP) [16]. The DV-hop method was first proposed by Dragos Niculescu at the University of Bucharest, and the basic idea is that one node estimates the distance to another node using the product of the network per-hop distance and the shortest path between them. The DV-hop algorithm does not need distance measurement equipment nor angle measurement hardware, and thus it is one of the most widely used algorithms for wireless sensor networks. It can be simply divided into three steps. First of all, the unknown nodes calculate the minimal hop counts to anchor nodes by the AODV (Ad hoc On-demand Distance Vector Routing) protocol. Then, the unknown nodes estimate their Euclidean distances to anchors by multiplying the minimum hop counts and the per-hop distance together. At last, the locations of unknown nodes are estimated by using the trilateration or maximum likelihood method. The Amorphous algorithm is an improved version of DV-hop. Unlike the first step used in DV-hop, Amorphous relies on another way that is called local hop counts to calculate the minimum number of hop counts. Furthermore, it also uses a different method to compute the average per-hop distance. In this way, Amorphous can generally achieve better performance than DV-hop. MDS-MAP, as another popular method, is a localization method that is based on multidimensional scaling (MDS). MDS-MAP uses the distance or connectivity information between all nodes at the same time and only uses the information between the unknown and anchor nodes. It can be used as either a range-based or a range-free algorithm. In the case of the range-free method, it rebuilds the relative coordinators of nodes by taking advantage of the pair distance, and obtains the absolute coordinators of nodes by using coordinate transformation with the help of anchor nodes. The above localization methods all assume that the nodes are distributed uniformly and densely in networks. However, the real deployment environment is often complex and changeable; thus, it is almost impossible to satisfy the ideal condition of uniformity and denseness for nodes to be located. For example, in anisotropic networks, such as networks with existing obstacles or noise corruption, these multi-hop range-free localization methods will probably demonstrate poor positioning accuracy.

Recently, designing localization algorithms through machine learning theory has drawn a lot of attention. These machine-learning-based localization algorithms utilize the correlations between nodal distribution characteristics and measurement information to construct a mapping model, which is then applied to estimate the positions of unknown nodes. Compared with the existing methods, machine-learning-based approaches can more effectively retrieve the fuzzy relations that are hidden behind information and data. Lim et al. proposed a localization method named Proximity-Distance Map (PDM) [17], which creates the mapping relationship between hop counts and the real distances of anchor nodes. Then, this mapping relationship is used to estimate the distance from the unknown nodes to the anchor nodes. Inspired by the PDM method in [17], Lee et al. presented two improved localization methods, i.e., Localized Support Vector Regression (LSVR) and Multi-dimensional Support Vector Regression (MSVR) [18], which regard the relationship between hop counts and distances as nonlinear and estimate distances by the regression method relying on a Support Vector Machine (SVM) [19].

Although the methods mentioned above can effectively improve the positioning accuracy in anisotropic networks, the computational complexity of their core algorithms is very high, which will cause sensor nodes to fail or die earlier because such nodes will estimate the locations by themselves, and they are short of computation and memory resources as well. Moreover, the LSVR and MSVR methods have too many parameters to be considered, which leads to very complex models; thus, they are not suitable for practical application scenarios. Recently, we proposed a novel DV-hop algorithm based on Locally Weighted Linear Regression (LWLR-DV-hop), in which the kernel method was adopted to improve the localization accuracy by raising the weight of neighboring anchor nodes [20]. Although the kernel method used in [20] improved the performance of DV-hop remarkably, it needs to compute the threshold of the hop count and the kernel parameter *k*, which definitely gives rise to computation complexity. Unlike [20], our proposed method involves only one parameter, and, thus, it has less computation complexity and can be regarded as an improved version of LWLR-DV-hop.

## 3. The Proposed Localization Algorithm

In this section, we will describe LSRR-LA in detail. Firstly, we present the localization problem in Section 3.1 and then deduce a formulated mapping model. Secondly, we will demonstrate the three steps of LSRR-LA in Section 3.2. Finally, the pseudo-code of LSRR-LA is illustrated.

### 3.1. Problem Statement

Consider a two-dimensional region of space, where there is a sensor network given by a set of nodes *N* = {*N*_1_, *N*_2_, …, *N_m_*_+*n*_}, which consists of m anchor nodes and *n* sensor nodes. The coordinates of these nodes can be described with definition (1):

(1) $$cor(N_{p})={\left(x_{p},y_{p}\right)}^{T},for\;p=1,...,m+n.$$

In WSN *N*, the positions of *m* anchor nodes *N_i_* ∈ ***A***, ***A*** = {*N_i_*|*i* = 1, ..., *m*} are known, while the positions of the *n* sensor nodes *N_j_* ∈ ***B***, ***B*** = {*N_j_*|*j* = *m* + 1, ..., *m* + *n*} are unknown. In the case of multi-hop networks, the hop counts between pair nodes are already known. The Euclidean distance from node *N_i_* to *N_j_* (*i* ≠ *j*) can be presented by Equation (2):

(2) $$d_{ij}=\left\|cor(N_{i})-cor(N_{j})\right\|=\sqrt{{\left(x_{i}-x_{j}\right)}^{2}+{\left(y_{i}-y_{j}\right)}^{2}}.$$

The hop counts from node *N_i_* to *N_j_* (*i* ≠ *j*) can be expressed as *h_ij_*. Taking Figure 1a as an example, in such a multi-hop network *d_ij_* is proportional to *h_ij_*, i.e., *d_i__j_* ∝ *h_ij_*. Thus, the localization problem can be formulated as:

(3) $$\begin{array}{l} \mathrm{Estimate}\;\mathrm{cor}\left(N_{k}\right),\\ \mathrm{Given}\;\mathrm{cor}\left(N_{i}\right),\;d_{ij},\;\mathrm{and}\;h_{kt}, \end{array}$$

where *N_i_*, *N_j_* ∈ ***A***, *N_k_* ∈ ***B***, and *h_kt_* denotes the hop counts between node *N_k_* and node *N_t_* (*N_t_* ∈ ***A*** ∪ ***B***). Assume that the distances and hop counts from *N_i_* to other anchors can be represented by the vectors **y***_i_* and **x***_i_*, respectively. Our aim is to learn a function *f*: X↦Y from *m* training data ${\left\{(x_{i},y_{i})\right\}}_{i=1}^{m}$, where **x***_i_* ∈ **X** and **y***_i_* ∈ **Y**. The input space **X** (hops), is known as the dependent variable, and the output space **Y** (distances) is the independent variable. The function *f* is represented by a linear model

(4) $$f(x)=w^{T}x,$$

where **w** is a *m* × 1 vector that contains the coefficients of the linear function. Usually, linear least squares (LLS) is used to determine w. It uses the vertical distance between the observed values **y***_i_* and the predictions *f*(**x***_i_*), which are known as the residuals **r***_i_* = **y***_i_* − *f*(**x***_i_*) = **y***_i_* − **w**^T^**x***_i_*.

In the LLS case, the sum of the squared residuals is minimized, which in matrix form is

(5) $$S(w)={\left(y-Xw\right)}^{T}(y-Xw),$$

with **X** being the *m* × *m* design matrix that combines all of the hop-count vectors, and the *m* × 1 vector **y** combining the distance values:

(6) $$X=\left[\begin{array}{cccc} x_{1,1} & x_{1,1} & \cdots & x_{1,m}\\ x_{2,1} & x_{2,2} & \cdots & x_{2,m}\\ \vdots & \vdots & \ddots & \vdots \\ x_{m,1} & x_{m,1} & \cdots & x_{m,m} \end{array}\right],\;y=\left[\begin{array}{c} y_{1}\\ y_{2}\\ \vdots \\ y_{m} \end{array}\right],\;$$

where each row corresponds to one input/output example. The coefficients could be optimized by minimizing Equation (5) as follows:

(7) $$\hat{w}=\arg\underset{w}{\min}{\left(y-Xw\right)}^{T}(y-Xw).\;$$

The minimizer of the problem (7) is

(8) $$\hat{w}={\left(X^{T}X\right)}^{-1}X^{T}y.\;$$

Thus, to make a prediction for a novel input **x**^new^, we only need to know the model and its parameters **w**, which can be represented as

(9) $$\hat{w}=\arg\underset{w}{\min}{\left(y-Xw\right)}^{T}(y-Xw).\;$$

### 3.2. Localization Algorithm

The proposed LSRR-LA contains the following three parts: the measurement part, the training part, and the localization part.

**Part A (Measurement)**: Assuming that **h**_i_ = [*h*_1,*i*_, ..., *h_m_*_,*i*_]^T^, *i* = 1, ..., *m* is the hop-count vector from anchor *N_i_* ∈ **A** to the other anchors, we thus can describe the hop-count matrix of all anchors as **H**_1_ = [***h***_1_, ..., ***h****_m_*]. Accordingly, the Euclidean distance vector and matrix between anchors can be represented as **d**_i_ = [*d*_1,*i*_, ..., *d_m_*_,*i*_]^T^, *i* = 1, ..., *m* and **D**_1_ = [***d***_1_, ..., ***d****_m_*], respectively. For any sensor node *N_j_* ∈ **B**, it has a hop-count vector to all anchors, **h**_j_ = [*h*_1,*j*_, ..., *h_m_*_,*j*_]^T^, *j* = *m* + 1, ..., *m* + *n*. Consequently, matrix **H**_2_ = [***h****_m_*_+1_, ..., ***h****_m_*_+*n*_] indicates the hop-count vectors from all of the sensor nodes to all of the anchors.

On referring to Formula (10), we express the hop-count matrix **H** (or the distance matrix **D**) by splitting it into four parts. We denote by **H**_1_ (resp. **D**_1_) the *m* × *m* matrix for the anchors versus themselves, **H**_3_ (resp. **D**_3_) the *n* × *m* matrix for the anchors versus sensors, and **H**_4_ (resp. **D**_4_) the *n* × *n* matrix for the sensors versus themselves. It is easy to know that **H**_2_ = **H**_3_^T^, **D**_2_ = **D**_3_^T^.

(10) $$H=\left[\begin{array}{cc} H_{1} & H_{2}\\ H_{3} & H_{4} \end{array}\right]=\left[\begin{array}{cc} \begin{array}{cccc} h_{1,1} & h_{1,2} & \cdots & h_{1,m}\\ h_{2,1} & h_{2,2} & \cdots & h_{2,m}\\ \vdots & \vdots & \ddots & \vdots \\ h_{m,1} & h_{m,2} & \cdots & h_{m,m} \end{array} & \begin{array}{cccc} h_{1,m+1} & h_{1,m+2} & \cdots & h_{1,m+n}\\ h_{2,m+1} & h_{2,m+2} & \cdots & h_{2,m+n}\\ \vdots & \vdots & \ddots & \vdots \\ h_{m,m+1} & h_{m,m+2} & \cdots & h_{m,m+n} \end{array}\\ \begin{array}{cccc} h_{m+1,1} & h_{m+1,2} & \cdots & h_{m+1,m}\\ h_{m+2,1} & h_{m+2,2} & \cdots & h_{m+2,m}\\ \vdots & \vdots & \ddots & \vdots \\ h_{m+n,1} & h_{m+n,2} & \cdots & h_{m+n,m} \end{array} & \begin{array}{cccc} h_{m+1,m+1} & h_{m+1,m+2} & \cdots & h_{m+1,m+n}\\ h_{m+2,m+1} & h_{m+2,m+2} & \cdots & h_{m+2,m+n}\\ \vdots & \vdots & \ddots & \vdots \\ h_{m+n,m+1} & h_{m+n,m+2} & \cdots & h_{m+n,m+n} \end{array} \end{array}\right],\;D=\left[\begin{array}{cc} D_{1} & D_{2}\\ D_{3} & D_{4} \end{array}\right]\;$$

Note that **H**_1_, **H**_3_, and **H**_4_ are known, while **D**_3_ and **D**_4_ are unknown. The goal is to predict **D**_3_ (or **D**_2_) from **H** and **D**_1_.

**Part B (Training)**: Now, we have the input matrix denoted by **H**_1_ and the output matrix represented as **D**_1_, and then we can train our linear model proposed in formula (4). However, as a difference from (4), here the coefficient vector **w** becomes a matrix **W** with *m* × *m*, and the outputs are stored in the matrix **D**_1_. Thus, the LLS solution becomes

(11) $$\hat{W}={\left(H_{1}^{T}H_{1}\right)}^{-1}H_{1}^{T}D_{1}.$$

In Formula (11), the variation range of the distance matrix **D**_1_ is different from that of the hop-count matrix **H**_1_. If the raw data is used directly, the calculation results will be affected due to the different variation range. We can eliminate this impact by preprocessing operations, such as normalization, making **D**_1_ and **H**_1_ have the same scale and to be equally emphasized. In this paper, the normalized matrices of **H**_1_ and **D**_1_ are represented by ${\tilde{H}}_{1}$ and ${\tilde{D}}_{1}$, respectively.

When calculating the model parameter **W**, we use the data of anchor nodes. If we include more anchor nodes, we will fit the training data more accurately, which means that our model is capable of minimizing the training errors more effectively. However, the minimization of training errors is not our goal. We hope that the model can accurately estimate the locations of sensor nodes, that is, the model should have a generalization capability. With many anchors, fitting the full model without penalization will result in large prediction intervals, and an LLS regression estimator may not uniquely exist. In order to avoid over-fitting, it is necessary to add a regularization item or a penalty term to the model [21]. In addition, because the LLS estimates depend upon ${\left(H_{1}^{T}H\right)}^{-1}$, there can be problems in computing **W** if $H_{1}^{T}H$ were singular or nearly singular. In this paper, a term *k***I** (*k* > 0) is added to the matrix $H_{1}^{T}H$ for remedying this problem. The method is also called Tikhonov regularization, which is the most commonly used method of regularization of ill-posed problems. Then, the estimated **W** can be described as

(12) $$\hat{W}={\left({\tilde{H}}_{1}{}^{T}{\tilde{H}}_{1}+kI\right)}^{-1}{\tilde{H}}_{1}{}^{T}{\tilde{D}}_{1}.$$

Here, the matrix **I** is an identity matrix. With the regularization term, the model can effectively avoid over-fitting. The *k* in the formula is called the hyper-parameters, which is used to balance the training errors and the regularization items [22]. Considering that ${\tilde{H}}_{1}^{T}{\tilde{H}}_{1}$ will be an ill-conditioned matrix if $\left\|{\tilde{H}}_{1}^{T}{\tilde{H}}_{1}\right\|<0.01$, we simply set the value of parameter *k* to be 0.01 in the later simulation part.

**Part C (Localization)**: Now, the normalized hop-count matrix ${\tilde{H}}_{3}$ and the coefficient matrix $\hat{W}$ are used to estimate the normalized matrix ${\tilde{D}}_{3}$,

(13) $${\tilde{D}}_{3}={\tilde{H}}_{3}\hat{W}.$$

After obtaining the result of Equation (13), we can derive **D_3_** by using a reverse operation of ${\tilde{D}}_{3}$, and then the trilateration method or the maximum likelihood method is used to estimate the coordinates of unknown nodes [23].

The pseudo-code of LSRR-LA is illustrated in Algorithm 1.
**Algorithm 1**: LSRR-LA**input: H**: hop matrix of the anchors and sensors; **D**_1_: distance matrix for the anchors versus themselves; *k*: hyper-parameters.**output:**${\text{\{}{\hat{x}}_{j}\text{\}}}_{j=m+1}^{m+n}$: estimated locations of the sensors.1 Normalize matrix **H**_1_ and **D**_1_, output ${\tilde{H}}_{1}$ and ${\tilde{D}}_{1}$;2 Calculate the mapping matrix $\hat{W}$ by Equation (12);3 Normalize matrix **H**_3_, output ${\tilde{H}}_{3}$;4 Calculate matrix ${\tilde{D}}_{3}$ by Equation (13);5 Derive **D**_3_ by using a reverse operation of ${\tilde{D}}_{3}$;6 Estimate coordinates ${\text{\{}{\hat{x}}_{j}\text{\}}}_{j=m+1}^{m+n}$ by using maximum likelihood method.

With respect to step 6 in Algorithm 1, for specific details the reader can refer to [23].

## 4. Performance Evaluations

In this section, we will evaluate the performance of our LSRR-LA in a simulative and experimental way. First, in Section 4.1, we analyze the complexity of those algorithms that include LSRR-LA and other classical algorithms, such as DV-Hop, Amorphous, PDM, LSVR, and MSVR. Then, in Section 4.2, we use the performance metric ALE (Average Localization Error) to evaluate these algorithms in C-shaped, L-shaped, and D-shaped network topologies. Finally, in Section 4.3, the positioning accuracy of LSRR-LA is verified in an outdoor experiment.

### 4.1. Complexity Comparison

The complexity mainly includes two aspects, i.e., communication complexity and computation complexity. The LSRR-LA method is similar to the DV-Hop, Amorphous, MDS-MAP, PDM, and LSVR methods. Each node needs to calculate the hop counts by flooding to other nodes, so their communication cost is equal, which is about *O*(*n*^2^*m*). Here, *n* denotes the number of sensor nodes while *m* indicates the number of anchor nodes. DV-hop and Amorphous use the Least Squares (LS) method to estimate the locations of sensor nodes, so they require computation complexity of about *O*(*m*^3^) [24]. MDS-MAP is a centralized localization method, and its location process can be divided into three steps: constructing the global shortest paths, executing the MDS algorithm, and converting the relative coordinates to absolute coordinates. Accordingly, the computation complexity is *O*(*n*^3^), *O*(*n*^3^), and *O*(*m*^3^ + *n*), respectively. The PDM method uses TSVD to process data in advance, which has a computation cost of *O*(*m*^3^) [25]; then, it uses the LS method to continue calculating data, so the PDM method costs more than DV-hop and Amorphous do. LSVR and MSVR use regression methods based on SVM, which needs to solve a quadratic programming problem [26]; thus, its computational complexity is *O*(*m*^2^)~*O*(*m*^3^). LSRR-LA uses the Least Square Regularized Regression method, so the computation cost is *O*(*m*^3^). Furthermore, its computation cost can be reduced to *O*(*m*^2^log*m*) by using the method introduced in [27].

### 4.2. Simulation Results

We conduct simulations in MATLAB (Jinling Institute of Technology, Nanjing, Jiangsu, China, version R2016b) and compare the performances of our proposed method with those of the previous methods, including DV-hop, Amorphous, MDS-MAP, PDM, and LSVR. The communication range of all of the sensor and anchor nodes is identical, and these nodes are deployed in a 1000 m × 1000 m square region for all simulations. We assume two types of distribution: regular distribution and random distribution. In each type of distribution, we considered three types of networks: C-shaped, L-shaped, and D-shaped. The networks used in the following simulations are presented in Table 1. Specially, we use the symbol ‘*’ to indicate the anchor nodes and ‘o’ to denote sensor nodes.

All of the reported results are the average over 100 trials. We used the ALE to evaluate the performances of all of the compared methods. The definition of ALE can be briefly formulated as

(14) $$ALE=\frac{\sum_{i=1}^{N}LocationError_{i}}{N}\;$$

where *N* is the number of unknown nodes. The location error of sensor node *i* can be formulated as:

(15) $$LocationError_{i}=\frac{\left\|{\hat{x}}_{i}-x_{i}\right\|}{R}\times 100\%,$$

where x*_i_* and ${\hat{x}}_{i}$ denote the real and estimated location of node *i*, respectively. *R* is the communication range of nodes and ‖•‖ represents the Euclidean distance.

The assumptions and simulation parameters are listed in Table 2.

#### 4.2.1. Regular Deployment

In the case of regular distribution, the sensor nodes and anchors are deployed uniformly along with the grids within the network. The size of the grid is set to 45 m × 45 m. We consider five levels of communication range *R*: 50 m, 100 m, 150 m, 200 m, and 250 m. Table 3 shows us the ALE results of the C-shaped network with 44 training anchor nodes. For example, the result of DV-hop is 283.8/92.6% in the case of *R* = 100 m, which means that the ALE of DV-hop is 283.8 and LSRR-LA improves the ALE by 92.6%. The ALE value 283.8 is a percentage, and if we turn this value to an absolute form, it will be 283.8 m (i.e., R*ALE/100). By contrast, the ALE value of LSRR-LA is 21.0, which means the absolute localization error is 21.0 m.

We can see from the simulation results in Table 3 that, as the communication range becomes larger, all of the competing methods demonstrate a performance improvement. However, all of the methods exhibit a substantial performance improvement except the proposed LSRR-LA method, which keeps a lower ALE as the communication range *R* increases gradually. It is clear that the proposed LSRR-LA method outperforms the previous methods, for it is rather stable and not sensitive to communication range.

We also considered five different anchor populations: 13.7% of the total nodes (*M* = 30 anchors), 18.3% (*M* = 40 anchors), 22.8% (*M* = 50 anchors), 27.4% (*M* = 60 anchors), and 31.9% (*M* = 70 anchors). Table 3 gives the ALE results of the C-shaped topology when the communication radius *R* = 100 m.

From the Table 4, it is obvious that the proposed LSRR-LA greatly outperforms the previous methods for the C-shaped anisotropic network. All of the competing methods demonstrate a performance improvement as the network gets denser; however, only the proposed LSRR-LA exhibits a substantial performance improvement while the previous methods do not exhibit satisfactory improvement.

**Figure 2:**
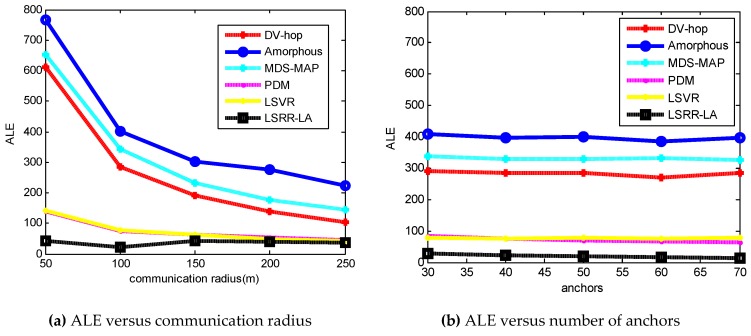
The ALE results of C-shaped regular networks.

Figure 2 shows a plot of Table 3 and Table 4. Figure 2a depicts the ALE results in terms of Table 2, where the communication radius varies from 50 m to 250 m. All of them generally conform to the trend that ALE decreases as the communication radius increases. When the communication radius *R* is smaller (*R* = 50 m), the positioning accuracy of DV-Hop, Amophos, and MDS-MAP becomes very poor. As the *R* increases, their localization errors begin to decrease. The LSRR-LA method always maintains the best positioning accuracy, whether *R* is smaller (*R* = 50 m) or *R* is larger (*R* = 250 m). Figure 2b shows the ALE results according to Table 4 where the number of anchor nodes increases from 30 to 70. It can be seen that the ALE change ranges of these algorithms are not violent, and the trend of ALE decreases gradually as the number of anchor nodes increases. As a result, LSRR-LA exhibits the best performance.

**Figure 3:**
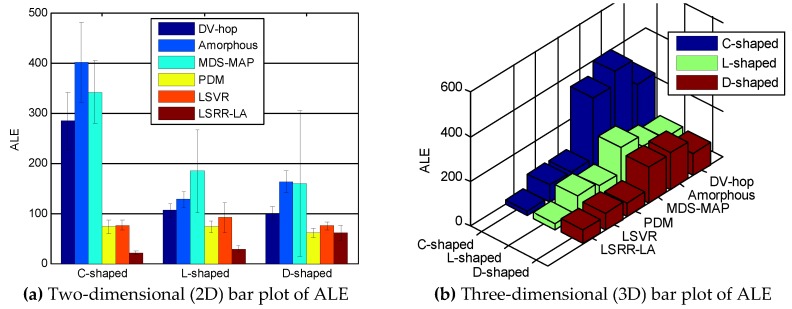
Comparison of ALE when *R* = 100 m and *M* = 20%.

Figure 3 shows an overall summarization of the six competing methods for the three anisotropic networks when *R* = 100 m and *M* = 20%. In contrast, LSRR-LA demonstrates the best positioning accuracy for the three different networks, and meanwhile keeps a small standard deviation.

**Figure 4:**
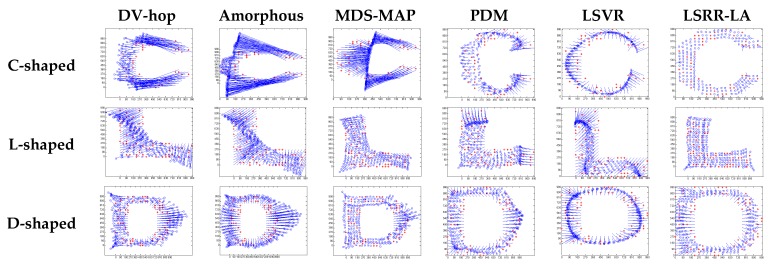
The localization results of the six algorithms in regular distribution networks (*R* = 100 m, *M* = 20%).

Figure 4 illustrates the localization results of the six methods and highlights the localization error of each node, when the communication radius *R* = 100 m and anchors *M* = 20% (under regular distribution). The blue circles indicate the estimated positions of the nodes and the blue lines describe the localization errors. The length of these lines is proportional to the localization errors.

**Figure 5:**
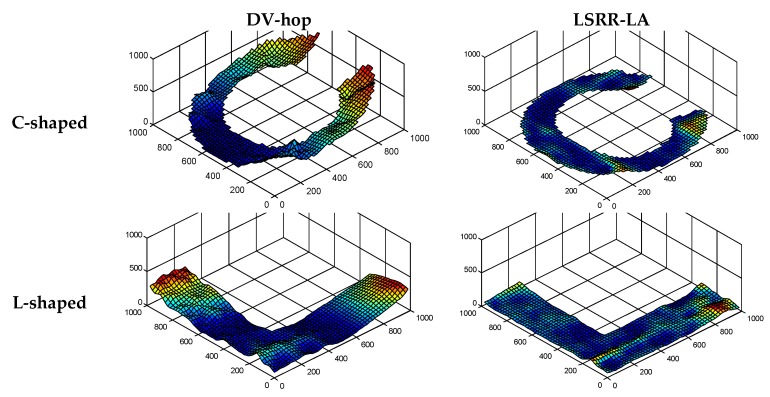

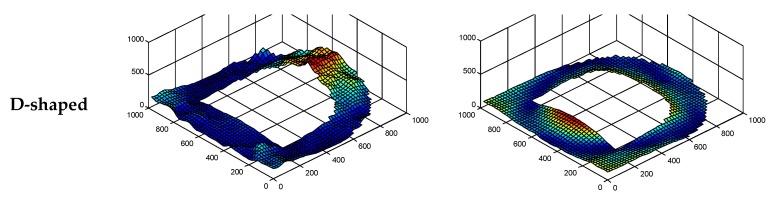
Comparison of the 3D Localization results (*R* = 100 m, *M* = 20%).

In addition, three-dimensional graphics are used to visualize the performance in order to show the localization results more clearly. In Figure 5, the three-dimensional (3D) localization results of LSRR-LA are compared with those of the classic DV-Hop method. The results prove that LSRR-LA outperforms DV-Hop, especially at the edge of these regions.

#### 4.2.2. Random Deployment

Sometimes, in the case of random deployment networks, some nodes cannot be connected to their neighbors due to the fact that the geometric distance between them may be beyond their communication range. This may cause a localization method to fail to estimate the locations of these isolated nodes, so we appropriately improve the total number of nodes and the communication radius to avoid this. Approximately 350 nodes (including anchor nodes and sensor nodes) are randomly distributed in an area of 1000 m × 1000 m. Table 5 shows the ALE results of the six methods when the total number of anchor nodes is 70 (20%) and the communication radius R varies from 100 m to 300 m.

We can see from the simulation results in Table 5 that, in the case of random deployment, our proposed LSRR-LA method achieves the best positioning accuracy. For example, when the node communication radius *R* = 200 m and anchors number *M* = 20%, the LSRR-LA method presents an ALE of 38.9, which is much better than the others.

As shown in Table 6, similar to regular deployment, five different anchor populations are also considered in random deployment. Here, we just show the ALE results of the C-shaped network when the communication radius *R* = 150 m and the number of anchors *M* is 65, 75, 85, 95, and 105, respectively. All of the competing methods demonstrate a performance improvement as the network becomes denser; however, these methods do not exhibit satisfactory improvements. Taking the LSRR-LA method for instance, we notice that its ALE is 38.9 with *M* = 65 while its ALE is only decreased to 34.8 with *M* = 105, which proves that anchor populations have just a little impact on positioning accuracy in random deployment networks, since it is not the same in the case of regular deployment networks (refer to Table 4).

**Figure 6:**
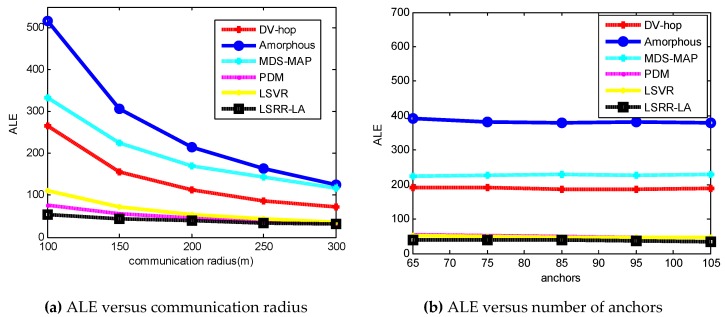
The ALE results of C-shaped random networks.

**Figure 7:**
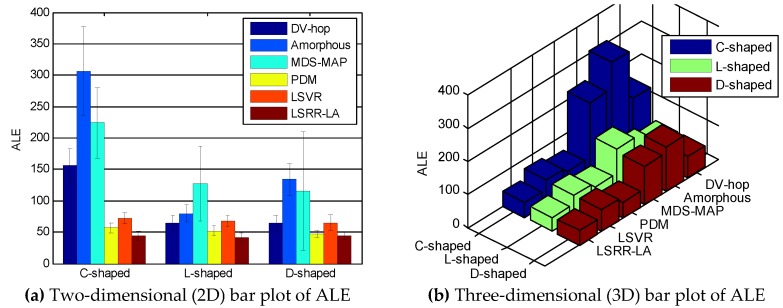
Comparison of ALE when *R* = 150 m and *M* = 70.

Figure 6 shows us the overall summarization of Table 5 and Table 6. Accordingly, Figure 6a depicts the ALE results of the six algorithms in random deployed networks, when the communication radius varies from 100 m to 300 m. The positioning accuracy of DV-Hop, Amophos, and MDS-MAP algorithm is very poor when the communication radius *R* is small (*R* = 100 m). Our LSRR-LA always maintains the highest positioning accuracy regardless of whether *R* is smaller (*R* = 100 m) or *R* is larger (*R* = 300 m). Figure 7b shows the ALE results of the six algorithms when the number of anchor nodes is increased from 65 to 105. We can see that LSRR-LA is still the best in these algorithms.

Similar to Figure 3, Figure 7 also shows an overall summarization of the six competing methods in random deployment networks, when the communication radius *R* is 150 m and the anchor node *M* is 70. It can be seen from the figure that our LSRR-LA demonstrates the best positioning performance.

**Figure 8:**
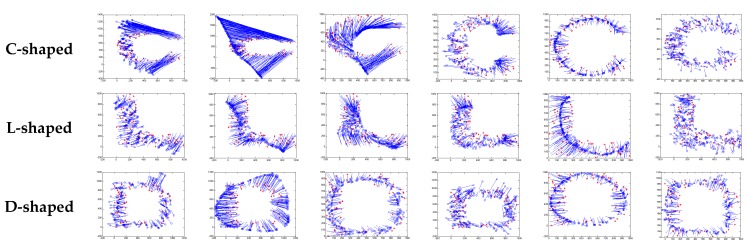
The localization results of the six algorithms in random deployment networks.

Figure 8 gives the localization results of the six algorithms when the communication radius *R* is 150 m and the number of anchors *M* is 70. Compared with Figure 4, LSRR-LA presents a rather stable performance regardless of whether it is in C-shaped, L-shaped, or D-shaped network; however, it is not the same in regular deployment networks, for it performs the best in the C-shaped network while it performs the worst in the D-shaped network.

**Figure 9:**
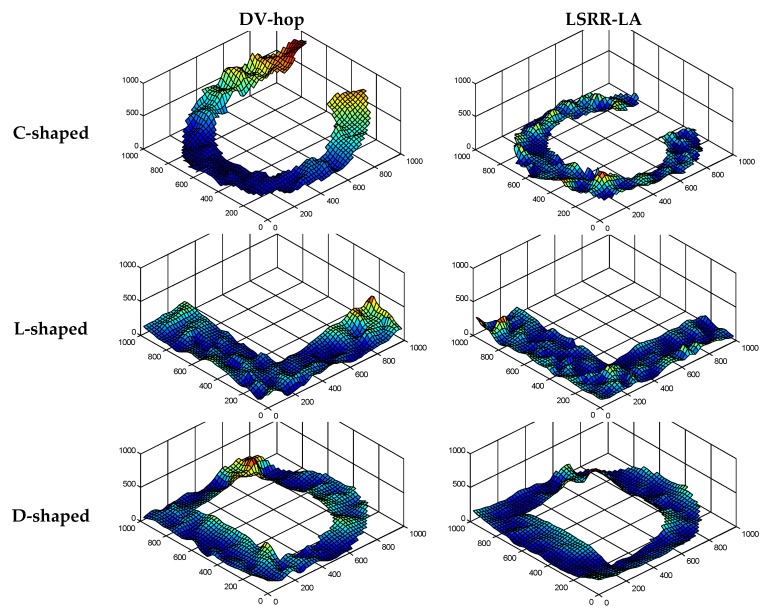
Comparison of 3D Localization results (*R* = 150 m, *M* = 70).

Similar to Figure 5, we also demonstrate the 3D localization errors in Figure 9 for a better comparison of positioning accuracy between LSRR-LA and DV-Hop. Our LSRR-LA still outperforms DV-Hop in the random deployment networks.

### 4.3. Experimental Results

**Figure 10:**
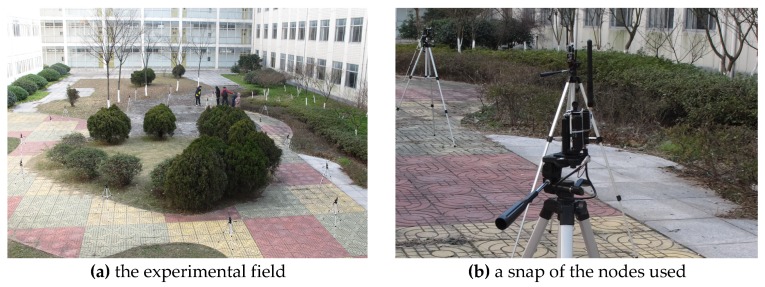
The real experimental scene.

In this section, we validate the performance of LSRR-LA through a practical experiment. As shown in Figure 10, a small vacant patch of land is chosen as the experimental field, which is about 25 × 25 m^2^. Some bushes, regarded as ‘obstacles’, are growing in the center of this field. A total number of 20 nodes were used in the experiment, and each node was mounted on a pole with a height of 1.3 m. All of the nodes are equipped with omnidirectional antennas and IEEE 802.15.4 compatible TI CC2450 transceivers operating in the 2.4 GHz ISM band.

**Figure 11:**
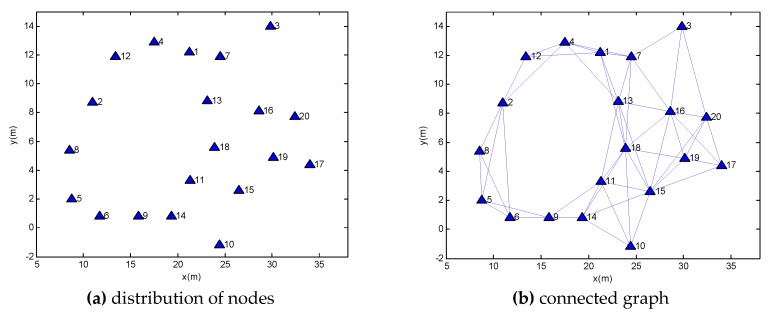
The network topology.

The distribution of nodes is shown in Figure 11a. We assign a number to each node. The transmission power is set to 0 dBm; thus, in this environment, each node can communicate range over a distance of 8 m. The connected graph of the entire network is shown in Figure 11b.

**Figure 12:**
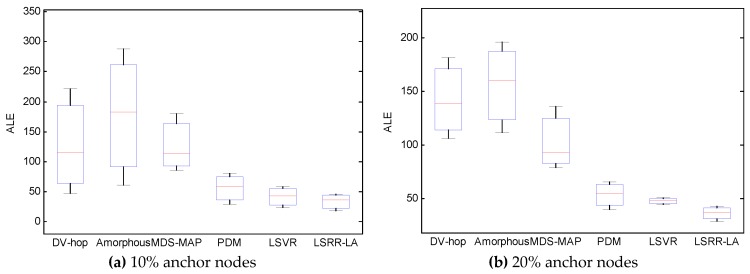

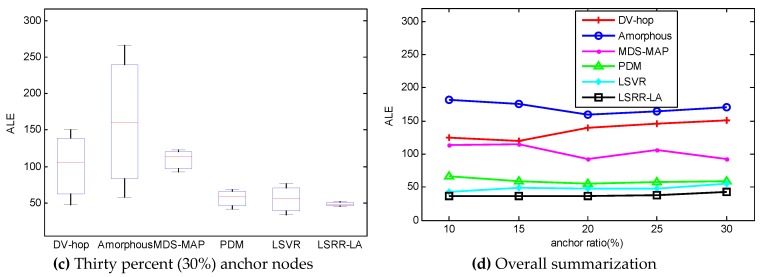
Comparison of the ALE under various anchor populations.

In the experiments, the anchor ratio is varied from 10% to 30%. The localization performance is compared using box plots in Figure 12. On each box, the central mark indicates the median while the bottom and top marks represent the minimum and maximum values, and the bottom and top edges of the box indicate the 25th and 75th percentiles, respectively.

Figure 12a–c show the localization results in which the anchor ratio is 10%, 20%, and 30%, respectively. The boxes represent the results of DV-hop, Amorphous, MDS-MAP, PDM, LSVR, and LSRR-LA, in that order.

From the figures, it is evident that the positioning accuracy depends proportionally on the number of anchor nodes for the compared localization algorithms DV-hop, Amorphous, MDS-MAP, PDM, LSVR, and LSRR-LA. It also proves that our proposed method has much better performance than the compared methods.

**Figure 13:**
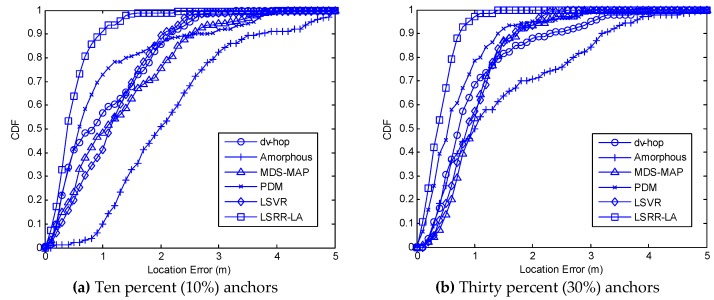
Comparison of the cumulative distribution function (CDF).

We also use the error cumulative distribution function (CDF) to represent the performance of our proposed algorithm, as shown in Figure 13. If anchor ratio is 10%, we can see from Figure 13a that the CDF is 63.8%, 85.5%, and 98.3% when the location error is 0.5 m, 0.8 m, and 1.5 m, respectively. If the anchor ratio is up to 30%, we can see from Figure 13b that the CDF is 67.1%, 92.8%, and 100.0% when the location error is 0.5 m, 0.8 m, and 1.5 m, respectively. As a comparison, the LSRR-LA performs best due to its advantage of the regression model.

## 5. Conclusions

In this paper, aiming at improving the positioning accuracy in anisotropic networks, we propose a localization method based on Least Square Regularized Regression, i.e., LSRR-LA, which considers the localization problem as a regression problem and constructs a mapping model between hop counts and Euclidean distance. By using this mapping model, we can estimate the locations of unknown nodes more accurately and more effectively. Compared with the localization methods DV-hop, Amorphous, MDS-MAP, PDM, and LSVR, our LSRR-LA outperforms them obviously, especially in anisotropic networks.

## Figures and Tables

**Table 1:** The simulation scenarios.

	C-Shaped	L-Shaped	D-Shaped
Random distribution	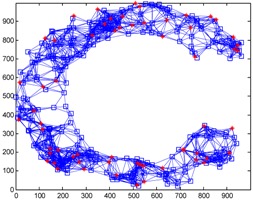	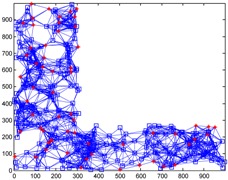	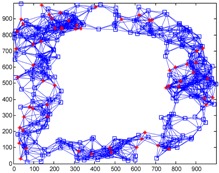
Regular distribution	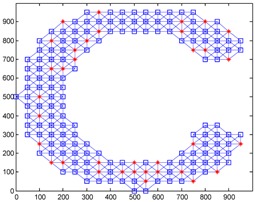	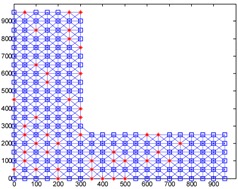	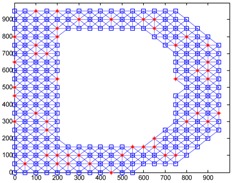

**Table 2:** The simulation parameters.

Parameter	Value
Compared methods	DV-hop, Amorphous, MDS-MAP, PDM, LSVR
Size	1000 m × 1000 m
Type of distribution	regular and random
Type of network	C-shaped, L-shaped, and D-shaped
Anchor node ratio (%)	13.7–31.9%
Communication radius (m)	50–300

DV-hop: Distance Vector hop; MDS-MAP: Multidimensional scaling MAP; PDM: Proximity-Distance Map; LSVR: Localized Support Vector Regression

**Table 3:** Comparison of the average localization error (ALE) under different radio ranges in the regular distribution network.

	*R* = 50 m/5%	*R* = 100 m/10%	*R* = 150 m/15%	*R* = 200 m/20%	*R* = 250 m/25%
Number of Anchors *M* = 44/20%					
DV-hop	613.1/93.2%	283.8/92.6%	189.9/78.4%	138.6/71.3%	103.9/65.6%
Amorphous	766.8/94.5%	400.4/94.8%	301.1/86.2%	275.7/85.6%	222.8/83.9%
MDS-MAP	651.8/93.6%	341.8/93.9%	231.1/82.2%	176.2/77.4%	143.8/75.1%
PDM	138.3/69.8%	73.5/71.4%	62.6/34.3%	53.4/25.6%	46.2/22.5%
LSVR	142.7/70.7%	76.2/72.4%	61.8/32.5%	49.3/19.4%	41.5/13.7%
LSRR-LA	41.8	21.0	41.9	39.7	35.8

LSRR-LA: Least Square Regularized Regression based multi-hop range-free Localization Algorithm

**Table 4:** Comparison of the ALE under different numbers of anchors in the regular distribution network.

	M = 30/13.7%	M = 40/18.3%	M = 50/22.8%	M = 60/27.4%	M = 70/31.9%
Communication Radius *R* = 100 m					
DV-hop	290.1/90.1%	285.2/92.2%	285.9/93.7%	271.4/94.2%	284.4/95.0%
Amorphous	408.1/92.9%	398.1/94.4%	400.4/95.5%	385.0/95.9%	398.4/96.45%
MDS-MAP	339.3/91.5%	329.7/93.2%	328.0/94.5%	331.8/95.2%	326.9/95.7%
PDM	85.6/66.4%	75.3/70.4%	68.1/73.7%	66.4/76.2%	63.5/77.7%
LSVR	78.4/63.3%	75.3/70.4%	77.1/76.8%	75.4/79.0%	78.4/81.9%
LSRR-LA	28.8	22.3	17.9	15.8	14.2

**Table 5:** Comparison of ALE under different radio ranges in the random deployment network.

	*R* = 100 m/10%	*R* = 150 m/15%	*R* = 200 m/20%	*R* = 250 m/25%	*R* = 300 m/30%
Number of Anchors *M* = 70/20%					
DV-hop	266.4/79.9%	156.3/71.6%	112.6/65.4%	87.4/61.3%	71.5/54.8%
Amorphous	516.2/89.6%	306.5/85.5%	215.5/81.9%	164.9/79.5%	125.6/74.3%
MDS-MAP	333.2/83.9%	224.2/80.2%	169.9 /77.1%	143.5/76.4%	117.3/72.5%
PDM	76.5/29.2%	56.8/21.9%	45.4/14.1%	37.9/10.7%	32.7/1.3%
LSVR	110.4/51.5%	71.9/38.3%	53.5/27.2%	42.8/21.0%	36.4/11.2%
LSRR-LA	53.5	44.4	38.9	33.8	32.3

**Table 6:** Comparison of ALE under different numbers of anchors in the random deployment network.

	M = 65/18.6%	M = 75/21.4%	M = 85/24.3%	M = 95/27.1%	M = 105/30.0%
Communication Radius *R* = 150					
DV-hop	192.2/79.7%	190.6/79.3%	186.4/79.5%	186.9/80.9%	188.2/81.5%
Amorphous	391.7/90.0%	382.4/89.7%	380.4/89.9%	382.0/90.6%	379.9/90.8%
MDS-MAP	225.4/82.7%	226.8/82.6%	230.2/83.4%	228.1/84.3%	230.6/84.9%
PDM	54.8/28.9%	52.1/24.4%	49.9/23.4%	47.8/25.2%	47.5/26.7%
LSVR	51.5/24.3%	49.0/19.7%	47.3/19.0%	46.8/23.7%	46.3/24.8%
LSRR-LA	38.9	39.4	38.3	35.7	34.8
